# Resorption Potential of Endoflas Powder With Curcumin Gel Against Endoflas for Pulpectomy in Primary Mandibular Molars: A One-Year Follow-Up Evidence-Based Case Reports

**DOI:** 10.7759/cureus.63231

**Published:** 2024-06-26

**Authors:** Pragyna Priyadarshini, Mahesh Ramakrishnan

**Affiliations:** 1 Paediatric and Preventive Dentistry, Saveetha Dental College and Hospitals, Saveetha Institute of Medical and Technical Sciences, Saveetha University, Chennai, IND

**Keywords:** pediatric endodontics, herbal medicine, postoperative pain, resorption potential, pulpectomy, primary teeth, obturating agent, eugenol, endoflas, curcumin

## Abstract

Pediatric endodontics in the primary teeth is chemomechanical due to the difficulties encountered in cleaning and shaping the root canals having complex anatomical configurations. Not only are obturating materials having ideal properties to be used in the primary teeth, but also imparting an impervious hermetic seal to prevent the nidus of reinfection is highly pivotal for the success of pulpectomy. However, certain obturating materials contain one or the other components that are irritant to the periapical region and aggravates the inflammatory process. Hence, a paradigm shift has been witnessed with the evolution of herbal medicines having innumerable beneficial properties with a broad-spectrum action to replace those components causing irritation and inflammatory reactions in conventional obturating materials. One such herbal medicament is “curcumin” popularly known as the “golden herb,” which has a wide repertoire of medicinal properties due to its bioactive component and volatile oil “turmerone.” Endoflas is one of the suitable obturating agents used in the primary teeth for its high success rate, but the use of eugenol in it is associated with irritation of the periapical region and causes necrosis of bone and cementum. Hence, the present case report aims to assess the rate of resorption, resorption potential, and the periapical healing of a novel obturating material Endoflas powder with curcumin gel (EPCG) replacing the liquid eugenol against conventional Endoflas material for pulpectomy in primary mandibular molars.

## Introduction

In India, the prevalence of dental caries in children below five years of age accounts for almost 48.11%, resulting in the early loss of the primary teeth [1]. Meticulous preservation of primary dentition is highly pivotal as natural teeth serve as the best space maintainer that aids in maintaining space for proper eruption of the succedaneous counterpart, intervening in any developing malocclusion, and facilitating the growth of the craniofacial complex [2]. Pulpectomy remains the mainstay of treatment in primary teeth with pulpal invasion. Nonetheless, it poses substantial treatment difficulties due to the complex anatomy of tortuous, ribbon-shaped, and blunderbuss canals; the proximity of the succedaneous tooth bud and multiple accessory canals close to the furcation area makes the biomechanical preparation highly challenging, resulting in the incomplete removal of microbes and eventually treatment failure [3].

To address this problem, obturating materials with high antimicrobial efficacy and possessing ideal characteristics should be used in the primary teeth [4]. Hence, Endoflas is one such material possessing the ideal properties of an obturating agent for the primary teeth. It comprises triiodomethane, zinc oxide (ZO), calcium hydroxide [Ca(OH)2], barium sulfate (BaSO4), iodine dibutyl orthocresol, and liquid components of eugenol and parachlorophenol [2]. Endoflas have the unique advantage of resorption limited to extra-radicular material without causing intra-radicular resorption [2]. However, its major disadvantage is eugenol as it is highly irritant to the periapical tissues, causes necrosis of the bone and cementum, and increases postoperative pain [5]. To overcome this problem, a revolutionary wave of using herbal medicines in pediatric endodontics has been introduced. One of the most studied medicinal herbs is “curcumin,” a rhizomatous herbaceous perennial plant, and its root (rhizome) has volatile oil “turmerone” and other coloring agents called “curcuminoids” [6]. The most significant curcuminoid is “curcumin,” and several studies have demonstrated its high anti-inflammatory, antimicrobial, and healing properties in periapical lesions of the oral cavity [7]. Therefore, curcumin gel can be a suitable alternative to eugenol in Endoflas for obturation in the primary teeth.

Hence, the present case report assesses the rate of resorption, resorption potential, and periapical healing potential of a novel obturating material Endoflas powder with curcumin gel (EPCG) against conventional Endoflas for pulpectomy in primary mandibular molars.

## Case presentation

Case 1

A seven-year-old female patient reported to the Department of Paediatric and Preventive Dentistry, Saveetha Dental College and Hospitals (SDCH), Chennai, India, with a chief complaint of night pain in the lower left and right back teeth region with difficulty in chewing food for three days. Clinical examination revealed deep dentinal caries with tenderness on percussion supplemented with radiographic findings of radiolucency involving enamel, dentine, and pulp in teeth 74 and 85 and furcation radiolucency measuring roughly 2-4 mm in tooth 85. A definitive diagnosis of chronic irreversible pulpitis was made for teeth 74 and 85. Pulpectomy was the recommended treatment modality using two different obturating materials, i.e., Endoflas in 74 and EPCG in 85. Each selected tooth was treated on different appointment dates to avoid high-intensity postoperative pain. Informed consent was obtained from the accompanying parent or caregiver. A topical anesthetic agent (Lignospan-O, Septodont Healthcare India, Pvt. Ltd.) was applied followed by the administration of local anesthesia using 2% lignocaine with 1:2,00,000 adrenaline (LOX* 2% ADRENALINE, Neon Laboratories Limited, India). Rubber dam (GDC Marketing, India) isolation was done. A no. 330 pear-shaped carbide bur (Mani, Inc, Tochigi, Japan) in a high-speed handpiece (NSK PANA AIR PA-SU B2) was used to remove superficial caries and to achieve a proper access opening. Coronal pulp amputation was done using a large round bur and a DG 16 endodontic explorer (Hu-Friedy Mfg. Co. LLC) was used to locate the root canals over the dentinal map. Radicular pulp was removed using a 10-size K-file (Dentsply Maillefer, OK, USA), and the remnant was removed using copious irrigation of normal saline (Fresenius Kabi India, Pvt. Ltd) in a 5 ml disposable syringe (Dispovan, Hindustan Syringes, and Medical Devices Pvt. Ltd).

Working length determination was done using pre-operative radiographs. The working length of each root canal was kept 1 mm short of the radiographic apex. Instrumentation of root canals was done using hand K-files. Frequent irrigation after each file instrumentation was done using 0.9% normal saline followed by 2% chlorhexidine (Asep- RC, Stedman Pharmaceuticals Pvt. Ltd, India). The shaped root canals were dried using no. 15-size sterile paper points (Pearl Dent. Co, Ltd., Vietnam), and obturation was done using Endoflas in 74. A powder-liquid ratio of 2:1 (Sanlor Laboratories, Colombia, USA) was dispensed on the glass slab using a measuring scoop. The powder was incrementally incorporated into liquid eugenol using an agate spatula (folding technique) to get the desired medium consistency. Similarly, 85 was obturated using EPCG with a powder-gel ratio of 3:1 (Curenext Oral Gel, Abbott Pharmacy, India, comprising *Curcuma longa *extract/rhizome (10 mg) with a gel base and available in paste form (50 gm)). A medium consistency was achieved by incorporating Endoflas powder incrementally into the curcumin gel using a stainless steel spatula (folding technique). The prepared material was then carried into the dried root canals in an incremental pattern using a no. 20-size handheld reamer (Mani, Inc., Japan). The final compaction of the material into the root canals was achieved by compressing the material into the canals using the wet cotton pellet technique. The coronal access cavity was restored using type II glass ionomer cement (GIC) (Shofu Inc., Japan) and an immediate postoperative intraoral periapical radiograph (IOPAR) was obtained to assess the quality of obturation. The final coronal coverage of the pulpectomized tooth was done using a stainless steel (SSC) metallic crown (3M ESPE) luted with type I GIC. All treatment procedures were performed by a single-trained oral pediatric clinician following all the standard treatment protocols. A similar procedure following the same protocol was performed by the same clinician on the other two patients mentioned below.

Follow-up

**Figure 1:**
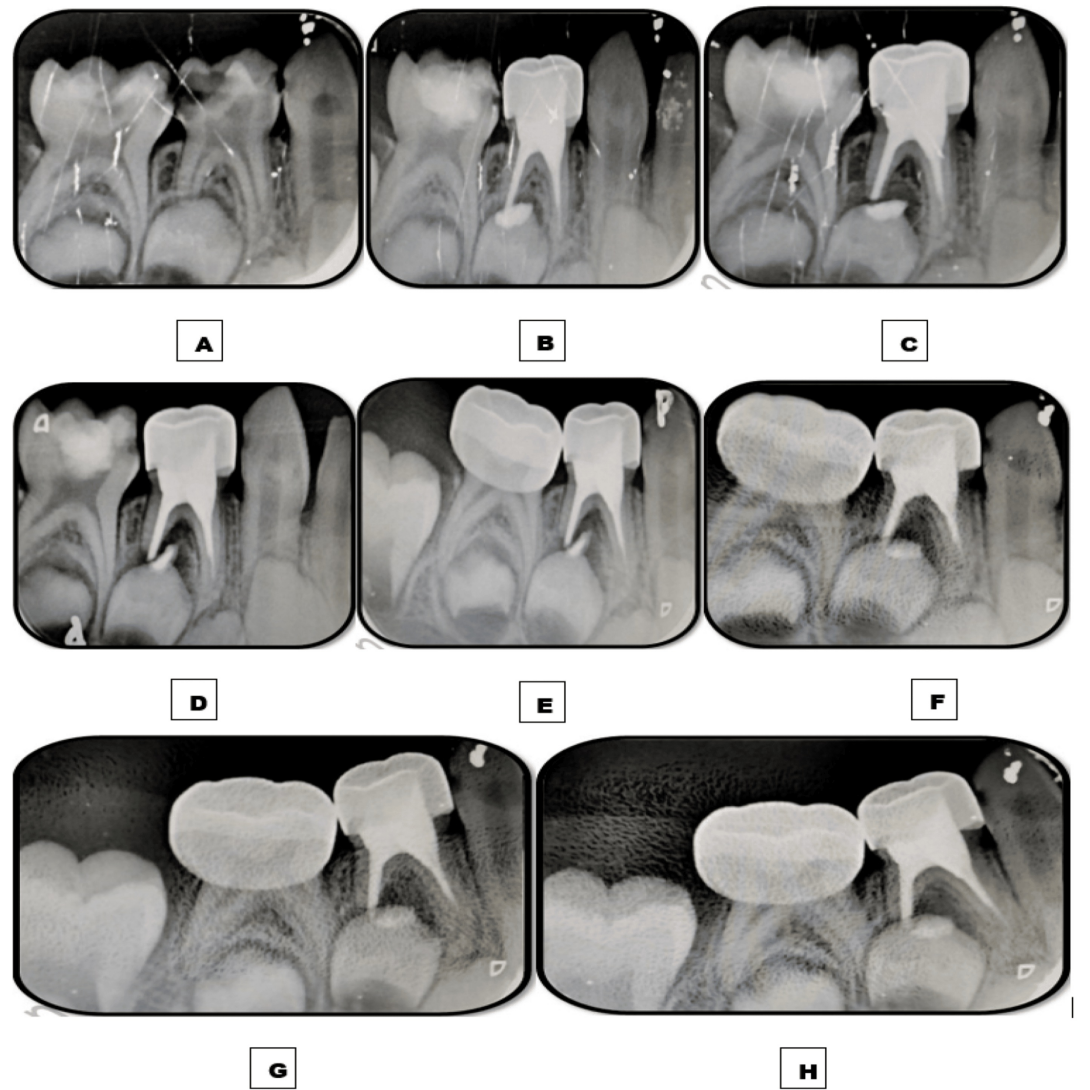
Images depicting the follow-up time interval of tooth 74 obturated with the Endoflas material. A: Preoperative immediate postoperative intraoral periapical radiograph (IOPAR). B: Immediate postoperative IOPAR depicting overfilled obturation. C: One-week follow-up IOPAR depicting the insignificant resorption of the apically extruded material. D: One-month follow-up IOPAR depicting no resorption of the extruded material. E: Three-month follow-up IOPAR. F: Six-month follow-up IOPAR. G: Nine-month follow-up IOPAR. H: 12-month follow-up IOPAR depicting the absence of resorption of the apically extruded material and no intra-radicular resorption of the obturating material.

**Figure 2:**
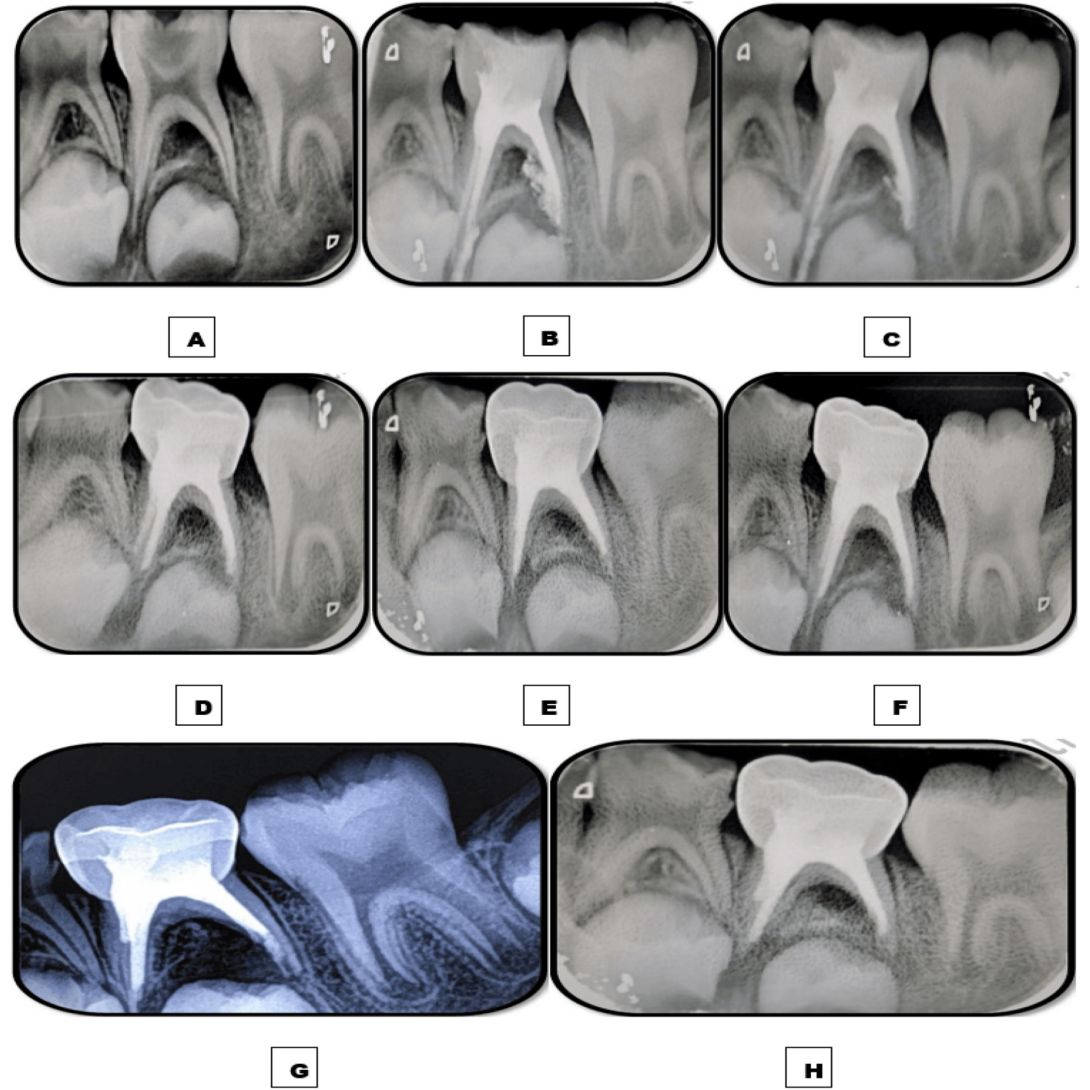
Images depicting the follow-up time interval of tooth 85 obturated with the Endoflas powder with curcumin gel (EPCG) material. A: Preoperative immediate postoperative intraoral periapical radiograph (IOPAR) depicting furcation radiolucency measuring roughly 2-4 mm. B: Immediate postoperative IOPAR depicting overfilled obturation. C: One-week follow-up IOPAR depicting marked resorption of the apically extruded material and significant resolution of furcation radiolucency. D: One-month follow-up IOPAR depicting the complete resorption of the extruded material. E: Three-month follow-up IOPAR. F: Six-month follow-up IOPAR. G: Nine-month follow-up IOPAR depicting the intraradicular resorption of one-third of the obturating material. H: 12-month follow-up IOPAR depicting no further intra-radicular resorption and significant resolution of furcation radiolucency.

Patient follow-up was done at an interval of one week and one, three, six, nine, and 12 months to assess alleviation of symptoms, rate of resorption and resorption potential of the obturating material, and resolution of furcation radiolucency. Postoperative IOPAR revealed overfilled obturation in both teeth (modified Coll and Sadrian criteria) [8]. Intermittent postoperative pain was experienced for two to three days after each pulpectomy and subsided on intake of analgesics. At one-week follow-up, insignificant extra-radicular resorption of Endoflas was observed in 74 while marked extra-radicular resorption of EPCG and significant resolution of furcal radiolucency was observed in 85. No further extra-radicular resorption was observed in 74 at follow-up intervals of one, three, six, nine, and 12 months, while complete extra-radicular resorption was observed in 85 at one-month follow-up. At the nine-month follow-up, intra-radicular resorption of one-third of the obturating material was observed in 85. At the 12-month follow-up, no further intra-radicular resorption was observed, and significant resolution of furcation radiolucency was seen in 85. No intra-radicular resorption was observed in 74 at the 12-month follow-up (Figures 1, 2).

Case 2

A five-year-old male patient reported to the Department of Paediatric and Preventive Dentistry, SDCH, Chennai, India, with a chief complaint of night pain in the lower left and right back teeth region with difficulty in chewing and food lodgement for one week. Clinical examination revealed grossly decayed teeth with tenderness on percussion supplemented with radiographic findings of radiolucency involving enamel, dentine, and pulp in the teeth 74, 75, and 84 and furcation radiolucency measuring roughly 2-3 mm in 74 and 84. A diagnosis of chronic irreversible pulpitis was made for 74, 75, and 84. Pulpectomy was done using EPCG in 74 and 75 and Endoflas in 84. The selected teeth in each quadrant were treated on the same appointment date and each quadrant was treated on different appointment dates. Similar treatment scheduling was also followed for the other patient mentioned below.

Follow-up

**Figure 3:**
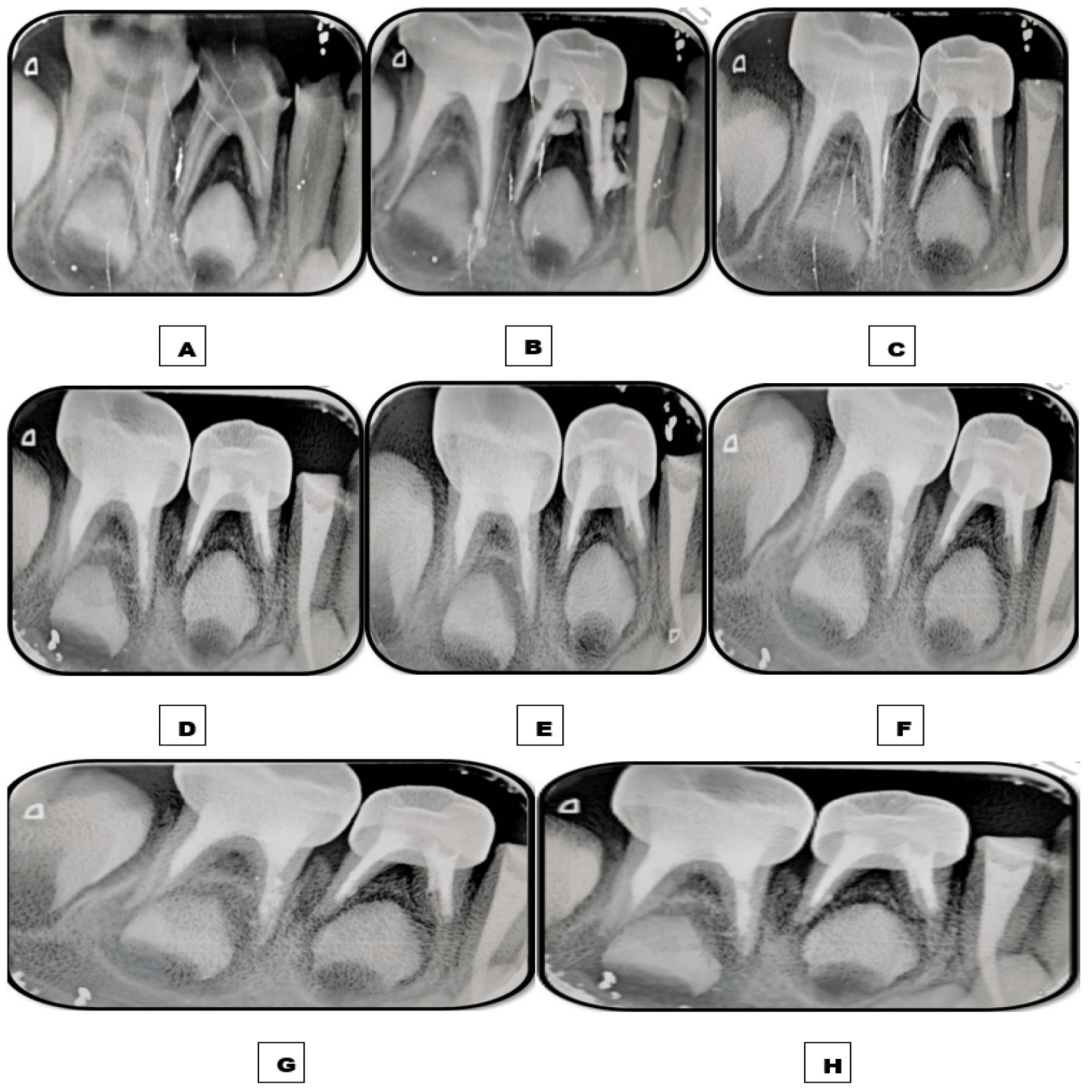
Images depicting the follow-up time interval of teeth 74 and 75 obturated with the Endoflas powder with curcumin gel (EPCG) material. A: Preoperative immediate postoperative intraoral periapical radiograph (IOPAR) depicting furcation radiolucency measuring roughly 2-3 mm in tooth 74. B: Immediate postoperative IOPAR depicting overfilled obturation. C: One-week follow-up IOPAR depicting the complete resorption of the apically extruded material in 74 and 75, and only a tinge of material remains unresorbed apically in 75. D: One-month follow-up IOPAR depicting the intra-radicular resorption of less than one-third of the obturating material in 74 and 75 and marked resolution of furcation radiolucency in 74. E: Three-month follow-up IOPAR. F: Six-month follow-up IOPAR. G: Nine-month follow-up IOPAR. H: 12-month follow-up IOPAR depicting no further intra-radicular resorption of the obturating material.

**Figure 4:**
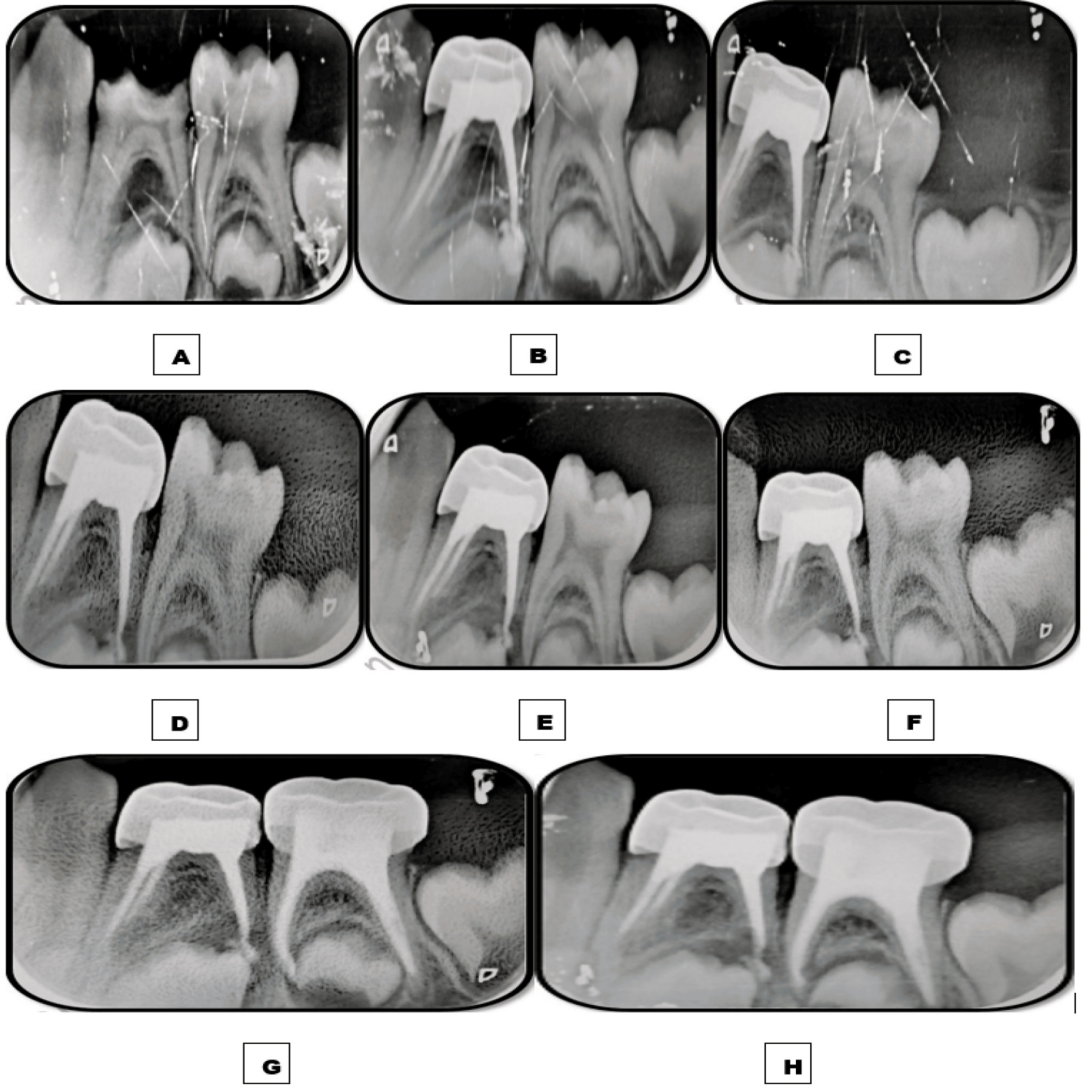
Images depicting the follow-up time interval of tooth 84 obturated with the Endoflas material. A: Preoperative immediate postoperative intraoral periapical radiograph (IOPAR) depicting furcation radiolucency of 2-3 mm. B: Immediate postoperative IOPAR depicting overfilled obturation. C: One-week follow-up IOPAR depicting no resorption of the apically extruded material and marked resolution of furcation radiolucency. D: One-month follow-up IOPAR. E: Three-month follow-up IOPAR. F: Six-month follow-up IOPAR depicting the significant resorption of the extruded material. G: Nine-month follow-up IOPAR. H: 12-month follow-up IOPAR depicting no intra-radicular resorption of the obturating material.

A similar follow-up interval was followed. Postoperative IOPAR revealed overfilled obturation in 74, 75, and 84. Intermittent postoperative pain was experienced for three to four days after pulpectomies in each quadrant and subsided by the end of one week on intake of analgesics. At one week follow-up, complete resorption of extra-radicular EPCG was observed in 74 and 75. By contrast, no resorption of extra-radicular Endoflas was observed in 84, but a marked resolution of furcation radiolucency was noted. At one month follow-up, intra-radicular resorption of less than one-third of the obturating material was observed in 74 and 75 with a marked resolution of furcation radiolucency in 74. At the six-month follow-up, significant extra-radicular resorption was observed in 84. By the end of the 12-month follow-up, no further intra-radicular resorption was observed in 74 and 75. A moderate amount of extra-radicular material did not resorb by the end of the 12-month follow-up without any evidence of intra-radicular resorption in 84 (Figures 3, 4).

Case 3

A six-year-old male patient reported to the Department of Paediatric and Preventive Dentistry, SDCH, Chennai, India, with a chief complaint of night pain in the lower left and right back teeth region with difficulty in chewing for five days. Clinical examination revealed grossly decayed teeth with tenderness on percussion and supplemented with radiographic findings of radiolucency involving enamel, dentine, and pulp in teeth 74, 75, 84, and 85. A diagnosis of chronic irreversible pulpitis was made for 74, 75, 84, and 85. Pulpectomy was done using EPCG in 74 and 75 and Endoflas in 84 and 85.

Follow-up

**Figure 5:**
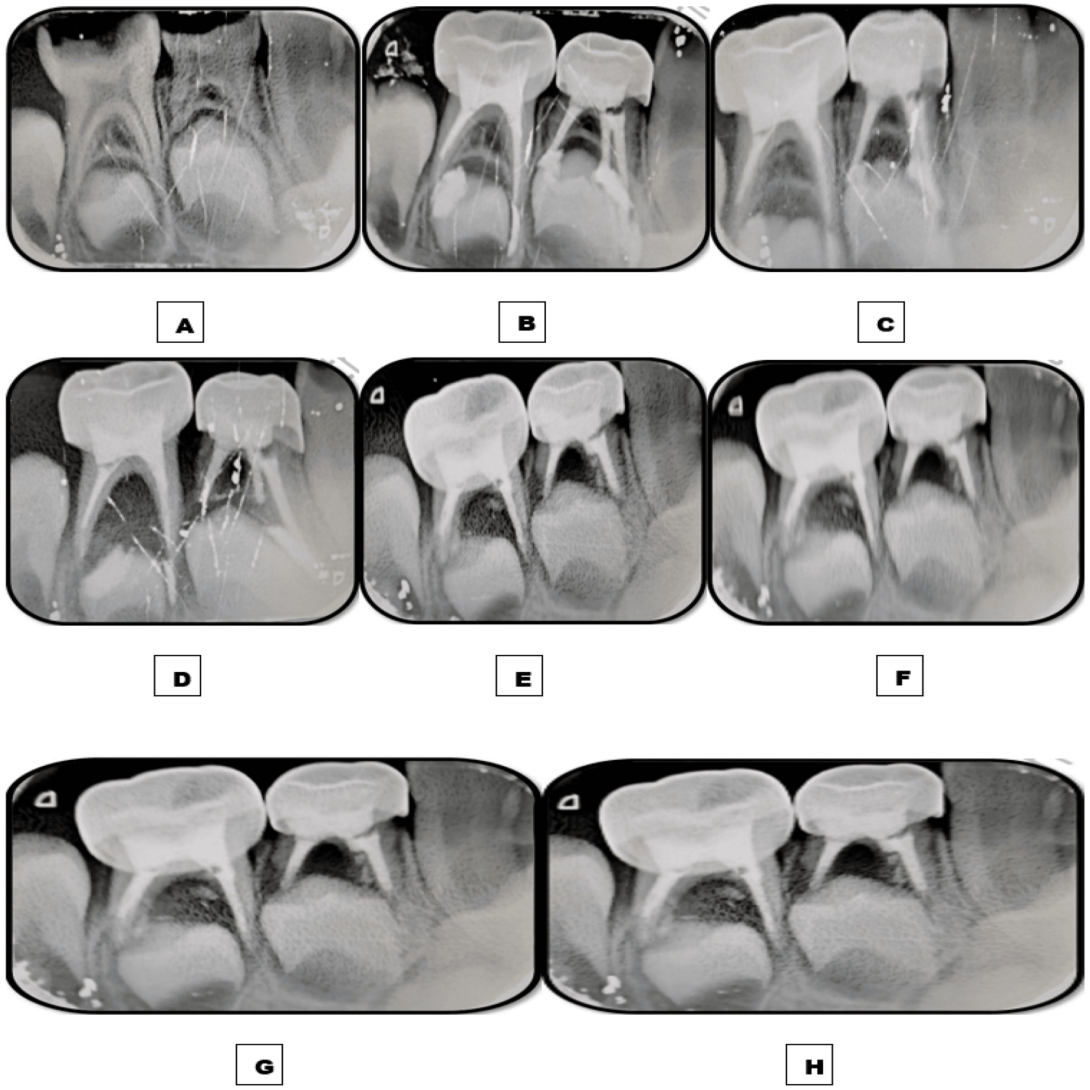
Images depicting the follow-up time interval of teeth 74 and 75 obturated with the Endoflas powder with curcumin gel (EPCG) material. A: Preoperative immediate postoperative intraoral periapical radiograph (IOPAR). B: Immediate postoperative IOPAR depicting overfilled obturation. C: One-week follow-up IOPAR depicting no resorption of the apically extruded material. D: One-month follow-up IOPAR depicting negligible resorption of the extruded material. E: Three-month follow-up IOPAR depicting complete resorption of the extruded material with a negligible intra-radicular resorption of the obturating material. F: Six-month follow-up IOPAR. G: Nine-month follow-up IOPAR. H: 12-month follow-up IOPAR depicting no further intra-radicular resorption.

**Figure 6:**
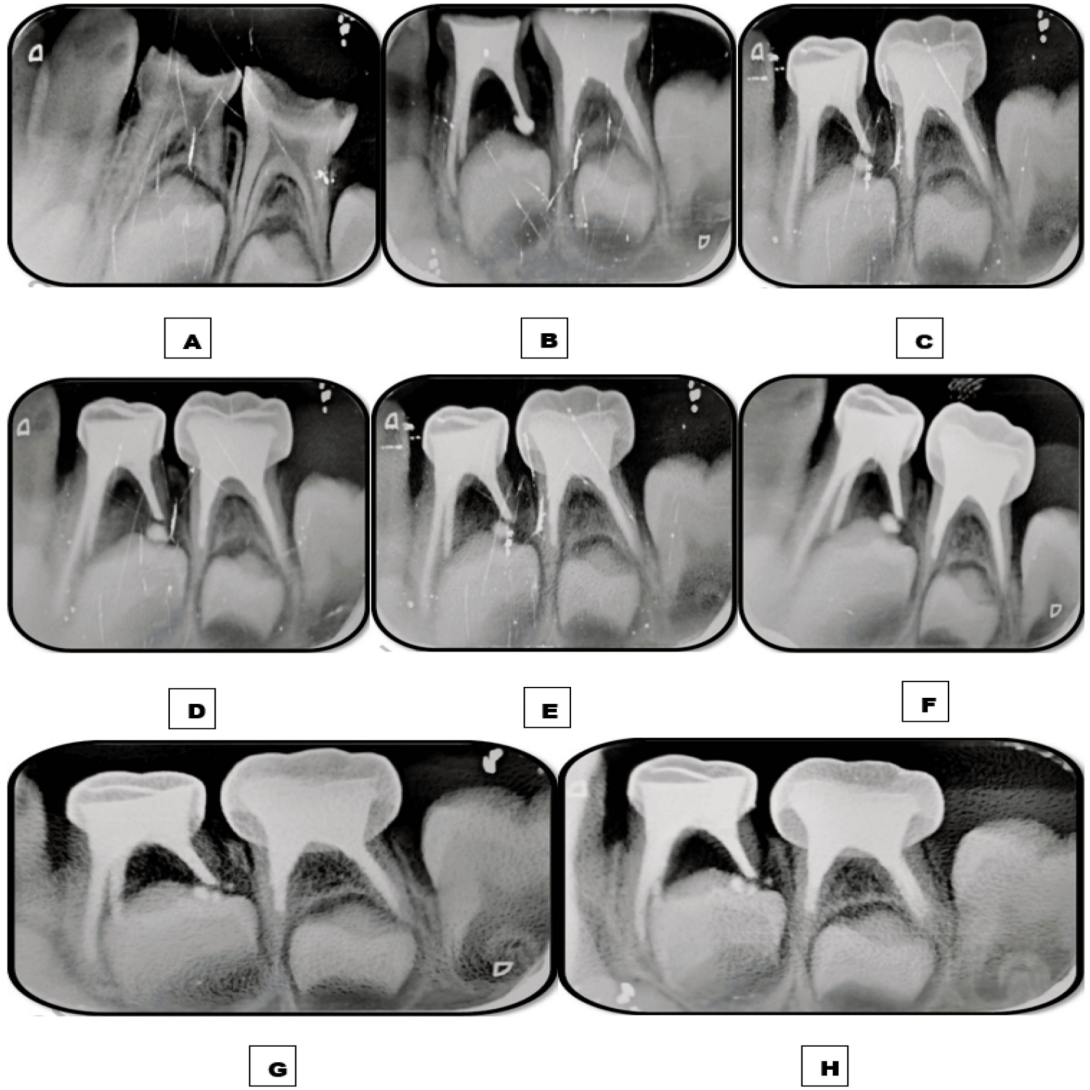
Images depicting the follow-up time interval of teeth 84 and 85 obturated with the Endoflas material. A: Preoperative immediate postoperative intraoral periapical radiograph (IOPAR). B: Immediate postoperative IOPAR depicting overfilled obturation in 84 and optimal filled obturation in 85. C: One-week follow-up IOPAR depicting no resorption of the apically extruded material in 84. D: One-month follow-up IOPAR. E: Three-month follow-up IOPAR. F: Six-month follow-up IOPAR. G: Nine-month follow-up IOPAR depicting negligible resorption of the extruded material in 84. H: 12-month follow-up IOPAR depicting no intra-radicular resorption of obturating material in 84 and 85 and incomplete resorption of extruded material in 84.

The same follow-up time interval was followed. Postoperative IOPAR revealed overfilled obturation in teeth 74, 75, and 84 and optimal-filled obturation in teeth 85. Intermittent postoperative pain was experienced for four to five days in teeth 74 and 75 and subsided by the end of a one-week follow-up on intake of analgesics. No extra-radicular resorption was observed by the one-week follow-up, while negligible extra-radicular resorption took place by the one-month follow-up in 74 and 75. At the three-month follow-up, complete extra-radicular resorption was observed in 74 and 75 with negligible intra-radicular resorption of obturating material. No extra-radicular resorption was observed till the six-month follow-up in 84, but negligible extra-radicular resorption was observed by the nine- and 12-month follow-ups. No further intra-radicular resorption was observed in 74 and 75 by the end of the 12-month follow-up. No intra-radicular resorption was observed in teeth 84 and 85, and incomplete extra-radicular resorption took place in 84 by the end of the 12-month follow-up (Figures 5, 6).

## Discussion

Endoflas has become the choice of material for obturation in the primary teeth owing to its excellent clinical performance and high success rate. Endoflas have the unique advantage of resorption limited to extra-radicular material without causing any intra-radicular resorption. Hence, the material is neither resistant to resorption nor results in a hollow-tube effect [9]. Fuks et al. (2002), in their landmark study, stated that Endoflas gets resorbed only as an extra-radicular material without causing premature intra-radicular resorption and reported an overall success rate of approximately 70% [10]. Endoflas has proven its suitability as a successful obturating agent in the primary teeth due to its powerful antimicrobial efficacy, high pH, firm adherence of material in mild humid root canals, and effective disinfection of dentinal tubules [11]. However, the major disadvantage of Endoflas is its liquid eugenol, which causes periapical irritation, inflammatory reaction, and tooth discoloration, despite being known for its high antibacterial effects and excellent healing properties [12,13]. Therefore, herbal medicine serves as a suitable alternative for obturation in the primary teeth. Curcumin is one such herb possessing high antibacterial properties, effective against root canal pathogens especially *Enterococcus faecalis*, regulates inflammatory processes, and is safe to be used in a wide range of concentrations [14-16].

In the present case report, all three patients reported intermittent postoperative pain for three to five days, which subsided on the intake of analgesics, and the probable reason could be the preoperative pathological condition, pain, and tenderness, which would have been exaggerated with the overfilled quality of obturation. Similar findings were observed by Priyadarshini et al. (2021), stating that children having overfilled obturation experienced moderate to severe postoperative pain, which subsided on the intake of analgesics by the fifth day of one-week follow-up [17]. The author also stated that a higher intensity of postoperative pain was found to be associated with Endoflas powder with turmeric gel (EPTG) obturation than with Endoflas obturation. Two important factors outlined in the trial for such an association were recruiting more children with preoperative pathology and a higher percentage of the overfilled obturation in the EPTG group [17]. However, in the trial high intensity of postoperative pain in the EPTG group was reduced within two to five days due to faster resorption of the extra-radicular material and curcumin regulating the existing inflammatory condition [17], which is similar to the findings observed in the present case report. Menni et al. (2020) stated complete resorption of extra-radicular curcumin gel mixed with Endoflas powder (CGE) at the end of the one-month follow-up [18], which is similar to the findings observed in three teeth obturated with EPCG showing complete resorption of the extra-radicular material within one-week to one-month follow-up interval, but significant resorption of the extra-radicular material took place within the one-week follow-up. By contrast, the remaining two teeth obturated with EPCG showed complete resorption of the extra-radicular material within one to three months of follow-up. In the present case report, four teeth showed significant resolution of furcation radiolucency within one-week to one-month follow-up, while Menni et al. observed a complete resolution of inter-radicular radiolucency within one to three months of follow-up [18]. The trial also stated the occurrence of intra-radicular resorption of CGE in three teeth at the one-month follow-up and five teeth each at the three- and six-month follow-ups. No radiographic data of any progressive intra-radicular resorption were provided by the end of the six-month follow-up [18].

However, in the present case report, negligible intra-radicular resorption of less than one-third of the obturating material (EPCG) was observed in all five teeth between one to nine months of follow-up, but no further intra-radicular resorption was observed in those teeth by the end of 12-month follow-up. Faster resorption of EPCG limiting to the extra-radicular material is mainly due to a balanced combination of zinc oxide (ZO), calcium hydroxide [Ca(OH)2], iodoform, and curcumin gel, where each powder component compensates for the disadvantages associated with the other component [11]. The addition of curcumin gel imparts high antibacterial efficacy, regulates the inflammatory process, and has been assumed to aid in the resorption pattern of the material through phagocytosis in a controlled manner [15]. Significant resolution of furcation radiolucency can be achieved due to curcumin and its volatile oil turmerone, which, combined with Ca(OH)2, further accelerates the antibacterial effect against the residing pathogens. It is this unique combination of faster resorption of the extra-radicular material, potent antibacterial, and anti-inflammatory effect coupled with a controlled resorption pattern limiting to the extra-radicular material that results in the early alleviation of high-intensity postoperative pain and makes EPCG a suitable alternative to the traditional obturating materials available for pulpectomy in the primary teeth.

By contrast, Pandranki et al. (2018) observed that overfilled teeth with Endoflas showed resorption of the extra-radicular material within 20 days to 11 months of follow-up. However, the complete resorption of the extruded material took almost six to 11 months of follow-up. The author also stated that 4% of the teeth showed early intra-radicular resorption, 88% had intra-radicular resorption at the same pace as the root resorption, and 8% showed delayed intra-radicular resorption compared to the physiologic root resorption [19]. In the present case report, only one out of four teeth (three overfilled and one optimal filled) showed significant resorption of the extra-radicular Endoflas material at the end of the six-month follow-up, but complete resorption of the extra-radicular material was not achieved by the end of the 12-month follow-up. The remaining two overfilled teeth showed negligible resorption of the extra-radicular material by the end of the 12-month follow-up. The probable reason for the resorption pattern of Endoflas limited to the extra-radicular material may be due to the fixative effect of parachlorophenol on giant cells indirectly responsible for the resorption of the extruded material [19].

However, in the present case report, delayed and incomplete extra-radicular resorption was observed, and the possible reason for it might be the impaired phagocytotic activity due to the prolonged irritating effect of eugenol to remove the obturating agent [11]. None of the teeth had intra-radicular resorption by the end of the 12-month follow-up. Therefore, the findings of Endoflas obturation in the present case report are not in accordance with the findings of the clinical trial conducted by Pandranki et al. Intra-radicular resorption in the trial could be mostly due to the existing pathology of recruited teeth. Patients in the following trial did not experience any postoperative pain within two years of follow-up except two patients each by the end of the one- and two-year follow-ups. As mentioned earlier, the intensity of postoperative pain was less in children with Endoflas obturation than with EPCG. The important reasons that can be outlined for such a finding are the treatment of teeth with mild or no associated pathology, decreasing the effect of eugenol as an irritant, and significant resorption of extra-radicular material within an interval of 20 days to four months. Pandranki et al. (2018) recruited 17 patients in the Endoflas group with preoperative periradicular rarefaction. Out of which, 11 teeth showed an increased size of rarefaction, and only six teeth had decreased rarefaction with complete healing. The author cited the selection of teeth for treatment to be pivotal for assessing the success rate of the material, which correlated with the increased size of rarefaction in the teeth associated with pre-existing moderate to advanced pathological conditions [19]. Reddy VV and Fernandes (1996) stated that variation in the rate of failures could also be due to individual body resistance [20]. On the other hand, only one tooth in the present case report had mild preoperative furcation radiolucency measuring roughly 2-3 mm, and significant resolution was observed by the end of the one-week follow-up since the tooth was associated with mild pre-existing pathology.

In the present case report, other findings such as deviation in the path of eruption of the succedaneous tooth, internal and external resorption, and any untoward reaction to biological tissues were not observed. One major limitation in the present case report that would have confounded the assessment of furcation radiolucency was the variation in the angulation of radiographic exposure altering the contrast of the radiograph to assess the formation of the initial meshwork of the bone matrix on the resolution of the inflammatory process and infection in cases of inter-radicular rarefaction. Such discrepancy was observed since using a radiograph positioner is inconvenient and causes much discomfort in children.

## Conclusions

The future scope of research entails extensive pediatric trials with long-term follow-up to assess the efficacy of different herbal materials showing favorable outcomes of obturating agents in the primary teeth. Both the materials, EPCG and Endoflas, showed favorable outcomes by the end of the 12-month follow-up and can be a suitable alternative to the conventional obturating agents used in pediatric endodontics. However, a long follow-up of a minimum 18-24 months interval should be followed to make keen observations regarding further changes caused by the material and its interaction in the biological realm.
